# Diabetes susceptibility in ethnic minority groups from Turkey, Vietnam, Sri Lanka and Pakistan compared with Norwegians - the association with adiposity is strongest for ethnic minority women

**DOI:** 10.1186/1471-2458-12-150

**Published:** 2012-03-01

**Authors:** Anne Karen Jenum, Lien My Diep, Gerd Holmboe-Ottesen, Ingar Morten K Holme, Bernadette Nirmar Kumar, Kåre Inge Birkeland

**Affiliations:** 1 Department of Endocrinology, Oslo University Hospital, Aker, Oslo, Norway; 2 Department of General Practice, Institute of Health and Society, Faculty of Medicine, University of Oslo, Oslo, Norway; 3 Faculty of Health Sciences, Oslo and Akershus University College, Oslo, Norway; 4 Oslo University Hospital, Oslo, Norway; 5 Department of Community Medicine, Faculty of Medicine, University of Oslo, Oslo, Norway; 6 Norwegian University of Sport and Physical Education, Oslo, Norway; 7 Norwegian Centre for Minority Health Research, Oslo University Hospital, Oslo, Norway; 8 Faculty of Medicine, University of Oslo, Oslo, Norway

**Keywords:** Diabetes, Ethnicity, Adiposity, Socioeconomic position, Life course

## Abstract

**Background:**

The difference in diabetes susceptibility by ethnic background is poorly understood. The aim of this study was to assess the association between adiposity and diabetes in four ethnic minority groups compared with Norwegians, and take into account confounding by socioeconomic position.

**Methods:**

Data from questionnaires, physical examinations and serum samples were analysed for 30-to 60-year-olds from population-based cross-sectional surveys of Norwegians and four immigrant groups, comprising 4110 subjects born in Norway (n = 1871), Turkey (n = 387), Vietnam (n = 553), Sri Lanka (n = 879) and Pakistan (n = 420). Known and screening-detected diabetes cases were identified. The adiposity measures BMI, waist circumference and waist-hip ratio (WHR) were categorized into levels of adiposity. Gender-specific logistic regression models were applied to estimate the risk of diabetes for the ethnic minority groups adjusted for adiposity and income-generating work, years of education and body height used as a proxy for childhood socioeconomic position.

**Results:**

The age standardized diabetes prevalence differed significantly between the ethnic groups (women/men): Pakistan: 26.4% (95% CI 20.1-32.7)/20.0% (14.9-25.2); Sri Lanka: 22.5% (18.1-26.9)/20.7% (17.3-24.2), Turkey: 11.9% (7.2-16.7)/12.0% (7.6-16.4), Vietnam: 8.1% (5.1-11.2)/10.4% (6.6-14.1) and Norway: 2.7% (1.8-3.7)/6.4% (4.6-8.1). The prevalence increased more in the minority groups than in Norwegians with increasing levels of BMI, WHR and waist circumference, and most for women. Highly significant ethnic differences in the age-standardized prevalence of diabetes were found for both genders in all categories of all adiposity measures (*p *< 0.001). The Odds Ratio (OR) for diabetes adjusted for age, WHR, body height, education and income-generating work with Norwegians as reference was 2.9 (1.30-6.36) for Turkish, 2.7 (1.29-5.76) for Vietnamese, 8.0 (4.19-15.14) for Sri Lankan and 8.3 (4.37-15.58) for Pakistani women. Men from Sri Lanka and Pakistan had identical ORs (3.0 (1.80-5.12)).

**Conclusions:**

A high prevalence of diabetes was found in 30-to 60-year-olds from ethnic minority groups in Oslo, with those from Sri Lanka and Pakistan at highest risk. For all levels of adiposity, a higher susceptibility for diabetes was observed for ethnic minority groups compared with Norwegians. The association persisted after adjustment for socioeconomic position for all minority women and for men from Sri Lanka and Pakistan.

## Background

Ethnicity may be defined as the social group a person belongs to because of a shared culture, history, geographical origin, language, diet, physical, genetic and other factors [1]. It has been found to exert an important influence on cardiovascular disease (CVD) mortality and a wide range of risk factors for CVD, particularly insulin resistance and type 2 diabetes [2,3]. Low socioeconomic position (SEP) explains much of the excess CVD mortality and some of the excess type 2 diabetes prevalence in ethnic minority groups [3]. The impact of structural or individual SEP on (patho-)physiological processes may start early in life, through a clustering and cumulative effect of risk factors [4]. In Europe, a high prevalence of diabetes has repeatedly been found in South Asians, mostly from Pakistan, India and Bangladesh [3,5,6], in Caribbeans and other groups with ancestral origin from Africa [3], as well as in people with origin from Turkey, Morocco and Middle East countries [7]. Type 2 diabetes is diagnosed up to 10-15 years earlier in the first generation of immigrants from Asia, Middle East/North Africa compared to Norwegians [8]. The reasons for the increased susceptibility is so far not attributed to genetic differences per se, but may be found in the complex interplay between gene expression and early and later life exposures. Foetal growth restriction, low birth weight and a short adult stature increase the risk of type 2 diabetes, and stunting may serve as a marker of adverse environmental influences hampering growth, eventually over generations [9-11]. Mean birth weight still varies between countries around the world and between ethnic groups within Europe [12,13]. Early catch up growth, long viewed as an essential recovery from the deleterious effects of poor growth on development and health, is now recognized as a risk factor for insulin resistance, obesity and type 2 diabetes [14].

Along with the worrisome increase in obesity and type 2 diabetes in women in reproductive age in most countries, an increase in gestational diabetes mellitus is observed [15], mostly so in susceptible ethnic groups. Women with gestational diabetes are at high risk for type 2 diabetes [16]. Pre-gestational physical inactivity, obesity, type 2 diabetes and even mild gestational diabetes may increase the risk of macrosomia, foetal adiposity and future diabetes in the offspring [15,17-19].

Central, and especially visceral, fat has been found to play an active role in the pathogenesis of insulin resistance and possibly also in the development of atherosclerosis, partly due to its stimulus to low-grade inflammation [20]. Nevertheless, there is conflicting evidence whether measures of central fat as waist circumference (WC), waist-hip ratio (WHR) or waist-to-stature (body height) ratio (WSR) are better predictors of CVD and type 2 diabetes than BMI [21-26]. Results from studies of cross-sectional [21,23,25] and prospective designs [22,24,26] as well as in different ethnic groups [23], may diverge in this respect. Body composition differs by ethnicity, and Asians have been found to have a higher percentage of fat or a deficit of lean mass compared with Europeans for a given BMI [20,25,27]. Adiposity, hyperinsulinemia and the thin fat phenotype in Indians may be present at birth [28]. As the impact of adult adiposity on the risk for type 2 diabetes and CVD seems to be stronger, especially in South Asians, but also in other Asian populations [20], ethnic specific definitions of obesity have been proposed [29,30].

Today, the number of ethnic minorities in Europe with origin from Asia, Africa and South America is rapidly increasing. The prevalence of diabetes and the mean BMI in South Asian women of childbearing age in Norway is alarmingly high [6]. High BMI is also found in other ethnic minority groups [31]. A better understanding of the interplay between the most important risk factors is warranted to plan effective, culturally sensitive and evidence-based interventions in the most susceptible ethnic groups. The aim of this study was to 1) investigate the association between the adiposity measures BMI, WHR, WC and WSR and diabetes in immigrant groups from Turkey, Vietnam, Sri Lanka and Pakistan compared with Norwegians, and 2) take into account confounding by socioeconomic position (SEP).

## Methods

### Participants, materials and methods

In 2000-2002 two population-based cross-sectional studies, both approved by the Regional Ethics Committee for Eastern Norway and The Norwegian Data Inspectorate and described in detail elsewhere [31-33], were performed by the Norwegian Institute of Public Health. The Romsås in Motion Study invited all 30-67 year olds in two Eastern districts in Oslo and The Oslo Immigrant Health Study all subjects 31-60 years of age from Sri Lanka, Pakistan, Vietnam, Turkey and Iran living in Oslo. The invitation was based on information on country of birth, age and residential address from population registers provided by Statistics Norway, responsible for coordinating all official statistics in Norway, including surveillance of living conditions and the demographic transition. The immigrant groups included in this study were among the largest in Norway and had the longest history of residence at the time of the study, according to Statistics Norway. Some, like the Vietnamese and Tamils from Sri Lanka, came mainly as refugees, while others, from Pakistan and Turkey, were primarily seeking labour or family reunion. In Norway, the proportion of ethnic minorities is largest in the Eastern districts of Oslo.

In both surveys, data were collected from questionnaires with information on self-reported disease, health-related behaviours and SEP, translated to the relevant languages, physical examination including body height, weight, waist and hip circumferences, blood pressure and serum analyses, all performed according to established standards [32].

**Figure 1:**
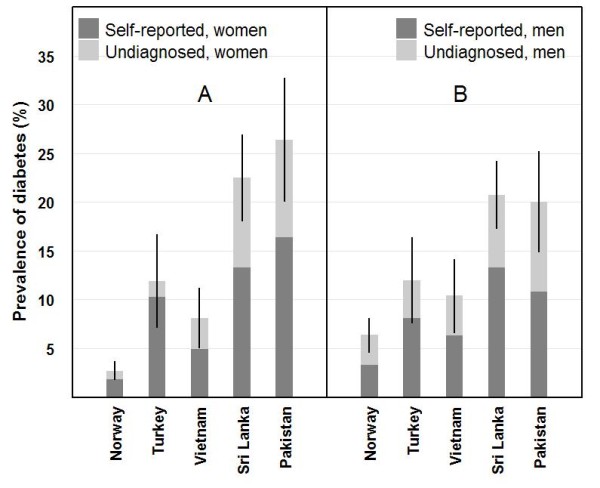
**Age standardized prevalence^a ^of self-reported and undiagnosed diabetes with 95% CIs (shown as lines) in women (A) and men (B) by country of birth**. ^**a **^The prevalences were standardized for age using the Norwegian population in 2000 as standard.

Ethnicity was based on country of birth, as immigration from the actual countries dates back only about three decades. For the immigrants, self-reported years of residence in Norway were recorded (more specifically time living in Oslo, recognized as a good proxy for time in Norway for the majority) [34]. Known diabetes was based on self-reports. Study subjects with non-fasting serum glucose (NFSG) levels > 6.0 mmol/l (measured by a Hitachi 917 auto analyzer, Roche Diagnostic, Switzerland) were requested to return within a few days for fasting serum glucose (FSG) and HbA_1_c (measured by HPLC (Variant, Bio-Rad, Richmond, CA, USA), normal reference range of 4.1-6.4%). Subjects not reporting diabetes, but with FSG ≥ 7.0 mmol/l, or HbA_1_c > 6.4%, or NFSG ≥ 11.1 mmol/l and not attending for fasting samples, were categorized as having undiagnosed diabetes (Figure 1). As The Romsås in Motion Study in 2000 revealed a high prevalence of self-reported and undiagnosed diabetes in all ethnic groups, re-invitation of those with (NFSG) levels > 6.0 mmol/l was also done in the Immigrant Health Study in 2002, after inclusion of the first 515 study participants. In the current study, the data from both studies were pooled to investigate the associations between anthropometry measures and diabetes in different ethnic groups, with Norwegians as reference.

**Figure 2:**
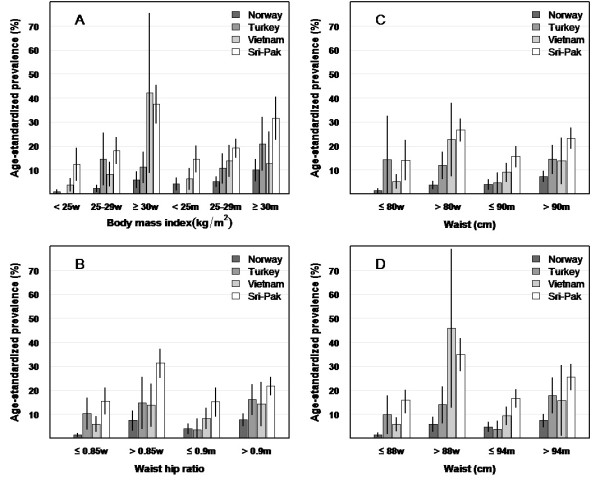
**Age-standardized diabetes prevalence^a ^by gender^b ^and country of birth for categories of the adiposity measures BMI (A), waist/hip-ratio (B) and waist (C and D)**. ^**a **^The age-standardized prevalences with 95% CIs for different adiposity categories were estimated by the direct standardization method (the command dstdize in Stata). ^b ^Gender w = women, m = men.

WC was measured with a measuring tape of steel at the midpoint between the iliac crest and lower margin of the ribs to the nearest 0.1 cm with the subject standing and breathing normally. Hip circumference was measured as the maximum circumference around the buttocks posteriorly and at the symphysis pubis anteriorly. We used ethnicity-specific definitions for overweight/obesity proposed by WHO: BMI ≥ 25/30 kg/m^2 ^respectively for subjects from Norway and Turkey, and BMI ≥ 23/25 kg/m^2 ^respectively for subjects from Sri Lanka, Pakistan and Vietnam (Figure 2) [30]. For WC we used the ethnicity-specific definition proposed by The International Diabetes Federation (IDF) [29] for men: 94 cm for subjects from Norway and Turkey, 90 cm for subjects from Sri Lanka, Pakistan and Vietnam. As IDF proposed a cut-off value of 80 cm for all ethnic groups of women, we added the Adult Treatment Panel (ATP) III definition of 88 cm to be applied for Norwegian and Turkish women [35]. For WHR we used cut-off values proposed by WHO: 0.85 for all women and 0.90 for all men [36]. Self-reported leisure-time physical activity was assessed by two validated questions on a four-graded scale [32]. In the analyses the variables were dichotomized (active versus sedentary) as very few were in the most active categories.

As SEP factors operating in different phases of the life course may influence diabetes risk, we wanted to include factors from early and later life [4]. The following indicators of SEP were used: Income-generating work, based on full-, part-time or no work participation (present SEP) [34] years of education (early adulthood SEP) [4] and body height (a proxy for childhood SEP) [37].

In The Immigrant Health Study, 3019 (39.7%) attended, 1006 (50.9%) from Sri Lanka, 448 (31.7%) from Pakistan, 537 (39.5%) from Vietnam, 426 (32.6%) from Turkey and 602 (38.5%) from Iran. In The Romsås in Motion Study, a total of 2593 subjects (49.0%) attended and were born in these five countries or in Norway, 2228 (49.5%) from Norway, 70 (64.2%) from Sri Lanka, 113 (41.7%) from Pakistan, 90 (45.5%) from Vietnam, 56 (40.0%) from Turkey and 36 (53.7%) from Iran. Of the 5612 subjects attending the two surveys, 360 subjects with age > 60 years (95% were Norwegians) were excluded to improve the comparability between the ethnic groups, as well as 683 due to other reasons (515 from The Immigrant Health Study included before the re-invitation procedures based on NFSG were established, 28 with only questionnaire data, 102 Romsås in Motion Study subjects who also attended the other study and 34 pregnant women) leaving 4569 subjects. Furthermore, the 459 Iranians were excluded due to few cases of diabetes (known diabetes: 12 subjects (women: 7/176, men: 5/283), survey-detected: 6 cases (all men)), leaving 4110 subjects as the study population. In all ethnic groups the participation rates among women were slightly higher than among men, except for Pakistanis where no difference was observed. Detailed analyses of the non-attendees have been performed for both studies [32,33]. In all ethnic groups the non-attendees had slightly lower education and income than the attendees.

### Statistical analyses

Proportions or percentages and means with 95% CIs were calculated for categorical and continuous variables. Adiposity variables and diabetes prevalences were age-standardized using the Norwegian population in 2000 and the direct standardization method. One-way analyses of variance, analyses of covariance and multiple logistic regression were used to calculate the *p*-values. (Table 1).

**Table 1:** Socio-demographic factors, body height and adiposity measures with 95% CIs by gender and country of birth

	Norway		Turkey		Vietnam		Sri Lanka		Pakistan		
	N = 1871		N = 387		N = 553		N = 879		N = 420		
	**Mean**^**a**^	95% CI	Mean	95% CI	Mean	95% CI	Mean	95% CI	%	95% CI	P*
Women											
Sample size (N)	1112		177		303		343		189		
Age (years)	46.3	45.8-46.8	41.7	40.7-42.8	43.4	42.5-44.2	39.6	38.9-40.3	43.5	42.4-44.6	< 0.001
Income-generating work - full time (%)	60.8	57.9-63.7	26.2	19.8-32.7	48.8	43.2-54.5	43.7	38.5-49.0	11.3	6.8-15.8	< 0.001
Self-reported years of education	12.2	12.0-12.4	6.0	5.3-6.8	10.3	10.0-10.6	12.1	11.7-12.4	8.4	7.6-9.1	< 0.001
Low education (% with ≤ 9 years)	17.7	15.5-19.9	70.6	63.9-77.4	43.6	38.0-49.1	12.8	9.3-16.4	43.1	36.0-50.2	< 0.001
Duration of residence in Norway (years)			16.0	14.4-17.5	11.9	10.7-13.0	11.4	10.3-12.5	16.5	15.0-17.9	< 0.001
Heavy physical activity, yes (%)	62.7	59.7-65.7	28.7	21.6-35.7	36.7	30.7-42.8	36.7	31.0-42.4	30.0	22.7-37.2	< 0.001
Body height (cm)	166.3	166.0-166.7	156.5	155.6-157.4	152.8	152.2-153.4	154.9	154.3-155.5	157.4	156.5-158.3	< 0.001
BMI (kg/m^2^)	26.1	25.8-26.3	31.7	30.8-32.6	23.4	23.0-23.7	27.1	26.7-27.5	29.6	28.8-30.3	< 0.001
BMI > 30 kg/m^2 ^(%)	19.1	16.7-21.5	55.9	48.6-63.3	4.9	2.4-7.3	22.4	18.0-26.8	41.2	34.2-48.3	< 0.001
BMI > 25 kg/m^2 ^(%)	50.3	47.3-53.2	87.7	82.9-92.6	25.1	20.3-30.0	66.4	61.4-71.4	82.1	76.7-87.6	< 0.001
BMI > 23 kg/m^2 ^(%)	70.2	67.5-72.9	96.2	93.4-99.0	48.9	43.3-54.6	85.2	81.4-88.9	92.6	88.9-96.4	< 0.001
Waist circumference (cm)	82.0	81.3-82.7	89.9	88.0-91.8	73.3	72.5-74.2	84.7	83.7-85.7	90.1	88.4-91.7	< 0.001
WHR	0.80	0.80-0.80	0.83	0.82-0.84	0.81	0.80-0.81	0.86	0.85-0.87	0.86	0.84-0.87	< 0.001
WSR^b^	0.49	0.49-0.50	0.57	0.56-0.59	0.48	0.47-0.48	0.55	0.54-0.55	0.57	0.56-0.58	< 0.001
Men											
Sample size (N)	759		210		250		536		231		
Age (years)	46.6	46.0-47.2	43.2	42.1-44.2	44.2	43.2-45.1	39.9	39.3-40.4	45.3	44.2-46.3	< 0.001
Income-generating work - full time (%)	87.4	85.1-89.8	54.3	47.6-61.1	64.1	58.1-70.0	71.1	67.3-75.0	61.2	55.0-67.5	< 0.001
Self-reported years of education	12.9	12.6-13.1	9.5	8.9-10.2	11.5	10.9-12.1	12.9	12.6-13.2	11.3	10.9-11.8	< 0.001
Low education (% with ≤ 9 years)	15.5	12.9-18.0	48.9	42.2-55.7	28.7	23.0-34.3	8.7	6.3-11.1	23.3	17.9-28.8	< 0.001
Duration of residence in Norway (years)			16.9	15.5-18.3	13.2	12.0-14.5	12.3	11.4-13.2	20.0	18.7-21.3	< 0.001
Heavy physical activity, yes (%)	67.6	64.1-71.0	33.7	26.9-40.4	45.5	38.8-52.2	51.9	47.1-56.7	33.0	26.4-39.6	< 0.001
Body height (cm)	179.1	179.3-180.3	170.6	169.7-171.	164.2	163.5-164.8	167.6	167.1-168.1	170.0	169.2-170.7	< 0.001
BMI (kg/m^2^)	27.1	26.8-27.4	28.0	27.5-28.5	24.1	23.8-24.5	25.9	25.6-26.1	27.4	27.0-27.9	< 0.001
BMI > 30 kg/m^2 ^(%)	19.6	16.8-22.4	27.4	21.4-33.4	2.7	0.7-4.7	9.3	6.9-11.8	23.2	17.8-28.7	< 0.001
BMI > 25 kg/m^2 ^(%)	68.5	65.2-71.8	79.8	74.4-85.3	36.1	30.1-42.2	59.6	55.4-63.7	75.8	70.3-81.4	< 0.001
BMI > 23 kg/m^2 ^(%)	87.5	85.1-89.8	90.2	86.2-94.2	66.6	60.7-72.5	81.8	78.6-85.1	88.7	84.6-92.8	< 0.001
Waist circumference (cm)	94.4	93.6-95.2	93.6	92.1-95.1	80.8	79.8-81.8	89.8	89.0-90.6	93.9	92.5-95.2	< 0.001
WHR	0.91	0.91-0.92	0.92	0.91-0.93	0.87	0.87-0.88	0.93	0.93-0.94	0.94	0.93-0.95	< 0.001
WSR^b^	0.53	0.52-0.53	0.55	0.54-0.56	0.49	0.49-0.50	0.54	0.53-0.54	0.55	0.54-0.56	< 0.001

^a ^Values are means unless stated otherwise. All variables (except age) are age-standardized for 30- to 60-year-olds with the Norwegian population in 2000 as standard

^b ^WSR: waist-to-stature (body height)-ratio

* P: *p*-values (one-way analyses of variance, analyses of covariance and multiple logistic regression were used to calculate the *p*-values.)

**Figure 3:**
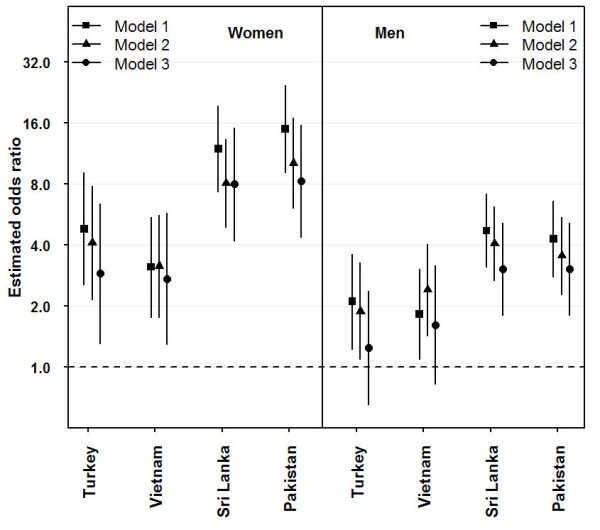
**Odds ratios (ORs) for diabetes with 95% CIs for ethnic minority groups compared with Norwegians from three models^a ^by gender**. ^**a **^Model 1: Adjusted for age, Model 2: adjusted for age and adiposity (WHR) and Model 3: adjusted for age, adiposity (WHR) and socioeconomic position (body height, education and income).

Separate analyses were performed for each gender since the Odd ratios (OR)s for diabetes in the ethnic groups were significantly different between men and women (*p *= 0.0003 for the interaction ethnicity/gender). The groups from Pakistan and Sri Lanka were merged in the analyses of associations for Table 2 and Figure 2 to condense the information, as they have a common origin in the Indian subcontinent. In Figure 3 the final multivariate results are presented for all four minority groups.

**Table 2:** ORs from logistic regression analyses with diabetes as dependent variable for ethnic minority groups versus Norwegians.

	Norway	Turkey	Vietnam	**Sri-Pak**^**a**^	*P*-value^b^
	OR (95% CI)	OR (95% CI)	OR (95% CI)	OR (95% CI)	
WOMEN					
Age (years)	1.77 (1.13-2.78)	4.31 (2.05-9.06)	2.75 (1.54-4.91)	2.00 (1.54-2.59)	0.166
Body height	0.48 (0.27-0.86)	0.77 (0.30-1.95)	2.21 (0.90-5.42)	1.10 (0.76-1.59)	0.031
BMI	1.63 (1.28-2.09)	0.94 (0.61-1.47)	2.72 (1.43-5.19)	1.63 (1.33-2.00)	0.068
WHR × 10	2.38 (1.69-3.36)	1.61 (0.76-3.41)	1.76 (1.05-2.95)	2.19 (1.64-2.93)	0.868
WC^c^	1.94 (1.44-2.63)	1.04 (0.60-1.82)	4.04 (1.94-8.40)	2.16 (1.64-2.85)	0.064
WSR^d^	2.00 (1.52-2.63)	1.06 (0.66-1.71)	2.89 (1.51-5.55)	1.93 (1.51-2.47)	0.134
Part/full time work					
Yes	1	1	1	1	
No	3.64 (1.82-7.29)	3.16 (0.64-15.63)	1.02 (0.39-2.67)	1.09 (0.69-1.73)	0.020
Education (years)					
> 9	1	1	1	1	
≤ 9	2.87 (1.39-5.94)	3.41 (0.41-28.63)	1.90 (0.72-5.05)	1.27 (0.75-2.16)	0.200
Heavy PA^e^					
Yes	1	1	1	1	
No	2.50 (1.16-5.37)	0.81 (0.24-2.71)	2.02 (0.62-6.61)	1.23 (0.73-2.07)	0.360
Parity 0-2	1	1	1	1	
3	1.27 (0.47-3.39)	10.51 (1.17-94.67)	0.85 (0.21-3.36)	0.71 (0.39-1.30)	
≥ 4	2.47 (0.71-8.59)	6.06 (0.68-53.82)	1.21 (0.39-3.74)	1.00 (0.57-1.75)	0.263
MEN					
Age (years)	2.12 (1.44-3.12)	3.06 (1.74-5.37)	2.26 (1.35-3.80)	2.59 (2.03-3.30)	0.709
Body height	0.79 (0.52-1.19)	0.92 (0.43-1.94)	0.88 (0.38-2.02)	0.86 (0.62-1.20)	0.989
BMI	1.58 (1.19-2.10)	2.70 (1.55-4.70)	3.41 (1.66-7.02)	1.55 (1.18-2.03)	0.074
WHRx10	2.00 (1.41-2.83)	2.56 (1.29-5.08)	2.44 (1.28-4.65)	1.61 (1.19-2.19)	0.507
WC^c^	1.81 (1.36-2.39)	2.56 (1.44-4.55)	2.66 (1.36-5.18)	1.46 (1.12-1.89)	0.173
WSR^d^	1.89 (1.43-2.52)	2.43 (1.40-4.23)	2.71 (1.42-5.18)	1.50 (1.17-1.93)	0.224
Part/full time work					
Yes	1	1	1	1	
No	2.28 (1.13-4.60)	2.40 (0.90-6.42)	2.57 (1.01-6.54)	1.37 (0.85-2.23)	0.489
Education (years)					
> 9	1	1	1	1	
≤ 9	2.36 (1.28-4.33)	1.14 (0.40-3.29)	1.31 (0.54-3.19)	0.65 (0.34-1.24)	0.058
Heavy PA^e^					
Yes	1	1	1	1	
No	1.38 (0.78-2.44)	1.41 (0.47-4.26)	2.47 (0.84-7.27)	0.96 (0.61-1.51)	0.404

All variables except age are age-adjusted

OR per SD for continuous variables (age, body height, BMI, WHR × 10, WC^c^, WSR^d^) and for comparing categorical variables with reference category

^a ^Sri Lankans and Pakistanis were merged to one group

^b ^test of interaction between ethnicity and risk factors on diabetes prevalence

^c ^WC: Waist circumference

^d ^WSR: Waist-stature (body height)-ratio

^e ^Heavy Physical Activity (≥ 1 hour per week versus no)

Age-standardized prevalences with 95% CIs for the different adiposity categories were estimated by the direct standardization method (the command dstdize in Stata). Continuous variables (adiposity measures, body height and age) were standardized by subtracting the population mean and dividing with the population SD (Table 2). Univariate and multivariate logistic regression models were used to estimate the ORs and 95% CIs for diabetes. WHR was multiplied by 10. Differences in age-standardized prevalences and age-adjusted OR of diabetes between the ethnic groups were tested by likelihood ratio tests (Figure 2, Table 2). We assessed whether the ethnic differences in OR for diabetes were significant after adjustments in multivariate models (Figure 3): Model 1: age, Model 2: age and adiposity (WHR) and Model 3: age, adiposity and SEP (body height, education and income-generating work), with Norwegians as reference. Possible two-way interactions were tested for significance on the relative scale. A significance level of 0.05 was used and two-sided *p*-values given. The analyses were performed in Stata 9.2 [38], R 2.8.1 [39] and SPSS 16.0 for Windows. The figures were made in R 2.8.1 [39] for Windows.

## Results

The main characteristics of the five population groups (N = 4110) are given in Table 1. The ethnic Norwegians were oldest and subjects from Sri Lanka youngest. Socioeconomic factors differed markedly between the ethnic groups, more for women than for men. Tamils from Sri Lanka had the lowest proportion with low education and subjects from Turkey the highest, and the latter had the lowest proportion with income-generating work. Ethnic minority men had longer duration of residence in Norway than women. Subjects from Pakistan and Turkey had longer residence than those from Vietnam and Sri Lanka.

Known diabetes was reported by 238 subjects (111 women/127 men), of these 18 (6.8%) were diagnosed before the age of 25 years. The total number with diabetes was 406 (176 women/230 men) when including the survey-detected cases constituting 37% of cases in women and 45% in men. The age-standardized diabetes prevalence (self-reported and total in Figure 1) and adiposity variables (Table 1) differed markedly between the ethnic groups. The highest prevalence of diabetes was found in subjects from Sri Lanka and Pakistan and the lowest in Norwegians. Subjects from Turkey had the highest BMI and the highest proportion with BMI ≥ 30 kg/m^2^, but subjects from Pakistan and Sri Lanka had the highest WHR and a substantially higher proportion with BMI ≥ 25 kg/m^2 ^and BMI ≥ 30 kg/m^2 ^compared to the Vietnamese. Nearly all women from Sri Lanka/Pakistan (85-92%) and 49% of women from Vietnam were overweight using the ethnic specific criteria (BMI ≥ 23 kg/m^2^), and the majority of female subjects from Sri Lanka/Pakistan (66-82%) but only 25% of female subjects from Vietnam were obese (BMI ≥ 25 kg/m^2^). Mean WSR was highest in subjects from Pakistan and Turkey and lowest in subjects from Vietnam. Applying the standard BMI definition for obesity (BMI ≥ 30 kg/m^2^) in the other female groups, 56% from Turkey and 19% from Norway were obese. All ethnic minority groups performed less heavy physical activity in leisure time, compared with Norwegians. No differences in age, years of education, self-reported diabetes or BMI were found between those included and the 515 subjects excluded from The Immigrant Health Study.

The age-standardized prevalence of diabetes was higher in the ethnic minority groups than in Norwegians for any level of BMI, WC and WHR when used as continuous variables except for men from Turkey in the lowest range of the adiposity variables (data not shown). When the adiposity measures BMI, WC and WHR were categorized (Figure 2), the age-standardized prevalence of diabetes increased more with increasing levels of these variables in ethnic minorities than in Norwegians. The age-standardized prevalences within each category of the adiposity measures were significantly different between the groups in both genders (all *p*-values < 0.001, likelihood ratio tests). Subjects from Norway with BMI ≥ 30 kg/m^2 ^had lower prevalence of diabetes than subjects from Sri Lanka and Pakistan with BMI 25-30 kg/m^2^. Lower prevalence in Norwegians was also found when applying higher cut-off values for WC for Norwegians (women: ≥ 88 cm, men: ≥ 94 cm) than for subjects from Sri Lanka and Pakistan (women: WC ≥ 80 cm, men: ≥ 90 cm). For the other ethnic groups the diabetes prevalence rates were higher than for the Norwegians in each adiposity category, but as there were few cases in some groups (Turkey: low adiposity groups, Vietnam: high adiposity groups), the confidence intervals were wide. However, the prevalence of diabetes in Vietnamese women was higher than in Norwegian women for all categories of WC and for those with WHR ≤ 0.85.

Before performing logistic regression analyses (Table 2, Figure 3), all continuous variables were standardized to allow for comparison. Overall, we found significant interactions between ethnicity and gender (*p *= 0.0003), and ethnicity and BMI (*p *= 0.017). In the gender specific logistic regression analyses adjusted for age, all adiposity measures were significantly associated with diabetes except in women from Turkey (Table 2). Ethnic differences in the OR for diabetes were only found for body height and income-generating work in women. As the age-adjusted OR for diabetes was highest for WHR in all ethnic subgroups except the Vietnamese, this anthropometric measure was used in the subsequent multivariate models.

We assessed whether the ethnic differences were significant after adjustments in Model 1: age, Model 2: age and adiposity (WHR) and Model 3: age, adiposity and SEP (body height, education and income-generating work), with Norwegians as the reference group (Figure 3). OR for diabetes was significantly increased for the four minority groups for both genders after adjusting for WHR and age. In women, the increased OR for diabetes persisted after further adjustment for all the SEP factors (Model 3) (Turkey: 2.9 (95% CI 1.30-6.36), Vietnam: 2.7 (1.29-5.76), Sri Lanka: 8.0 (4.19-15.14), Pakistan: 8.3 (4.37-15.58). No SEP variables were independently related to diabetes in women in Model 3.

In men, having no income-generating work was independently related to diabetes (OR 1.6 (1.10-2.27)) along with age (2.0 (1.59-2.40)), WHR (1.9 (1.49-2.29)) and ethnicity (see Figure 3). For men from Sri Lanka and Pakistan the increased OR compared with Norwegians persisted after adjusting for all SEP variables, with identical OR (3.0 (1.80-5.12)). For men from Turkey, however, when adding any of the SEP indicators into the model, the increased OR for diabetes compared with Norwegians was no longer significant. For men from Vietnam the increased OR for diabetes persisted when adding either income-generating work (1.8 (1.04-3.20)) or education (2.5 (1.47-4.23)), but when adding body height no ethnic difference was found (1.8 (0.96-3.40)).

Applying WC or WSR instead of WHR did not explain more of the ethnic differences, nor did adding any of the two physical activity variables, smoking, duration of residence in Norway and parity (for women) to the model. No significant interactions were found in the full multivariate model.

## Discussion

We found an alarmingly high prevalence of diabetes in the groups from Sri Lanka and Pakistan (20-26%) in Oslo. Higher diabetes prevalence rates were also found in women from Turkey and Vietnam compared with Norwegian counterparts. An increased susceptibility for diabetes in all ethnic minority groups was observed compared with Norwegians for the same categories of BMI, WC and WHR, highest for Sri Lankans, Pakistanis and Vietnamese, and more for women than men. The ORs for diabetes were highly significant for all ethnic minority groups compared with Norwegians when adjusted for age and WHR. After further adjustment for SEP from early and later life (body height, education and income-generating work), the ORs for diabetes were still significantly increased for all ethnic minority women and for men from Sri Lanka and Pakistan.

The diabetes prevalences for subjects born in Vietnam, and especially for those born in Sri Lanka and Pakistan, are higher than reported from their countries of origin, even from urban areas (Sri Lanka 2005/2006: urban 16%/rural 9%, Pakistan 1992-1996: urban 11%/rural 8%, Vietnam 2001: urban women 8%/urban men and rural men and women 5%) [40-42]. The diabetes prevalence for those born in Turkey were comparable with national data (Turkey 2000: men 12.9%/women 10.9%, no urban/rural difference) [43]. Our prevalence rates are probably underestimated as we did not perform OGTT due to resource limitations, and the proportion of undiagnosed subjects in our study was generally lower (Turkish women: 13%, other groups: 33-48%) than in most of the studies from Asia (36-60%) [40-43].

Furthermore, mean BMI and/or the proportion with BMI ≥ 23, 25 or 30 kg/m^2 ^were substantially higher for these ethnic minority groups living in Norway, especially among women, than in their countries of origin [40-43]. Studies of the same migrant groups from other European countries have repeatedly found diabetes prevalence in South Asians of about 20% [5] and in Turkish migrants comparable to our study [7]. However, mean BMI and/or proportion with BMI ≥ 25 or 30 kg/m^2 ^in the groups from Pakistan and Turkey living in Norway are higher than reported in these groups from most other European countries, especially in women. The real total diabetes prevalence in these groups in Norway may therefore be even higher than in other European countries.

As all adiposity measures predicted diabetes in all groups (except Turkish women), deposition of excess fat seems to be crucial regardless of ethnicity. WHR was the strongest predictor, except among the Vietnamese where WC (women) and BMI (men) were the strongest predictors. WHR includes another measure, hip circumference, also found to be inversely associated with diabetes in some studies [44]. In our population WSR did not improve the prediction of diabetes compared with BMI, WC and WHR. Although the amount of visceral fat can be more exactly quantified by CT or NMR than with WC or WHR, these measures are expensive, may infer exposure to radiation and are still not applicable in large-scale epidemiological studies [44]. The relevance of the simple measures of central fat in addition to BMI can be illustrated by our findings that the highest diabetes prevalence was found in subjects from Sri Lanka and Pakistan who had the highest WHR, although general obesity was most prevalent among subjects from Turkey who had lower diabetes prevalence. Worth to note is that women from Vietnam with a mean BMI of 23.4 kg/m^2 ^seem to store relatively more fat centrally compared with their Turkish and Norwegian counterparts and have a markedly increased risk of diabetes. WHR was positively associated with high-fat foods, inversely associated with degree of integration and was not associated with duration of residence in South Asians living in Norway [34]. However, even when applying ethnicity-specific definitions for overweight and obesity proposed by WHO [30] or comparing different cut-off values for WC, the risk for diabetes in women from Sri Lanka, Pakistan and Vietnam were still higher than in Norwegian and Turkish women. Most studies aiming to identify the optimal cut-off points for the obesity measures use receiver operating characteristics and logistic regression analyses. In a study using principal component factor analyses, a BMI of 30 kg/m^2 ^in Europeans was found to be equivalent to a BMI of about 21 kg/m^2 ^in South Asians and Chinese populations with respect to the risk of diabetes, also indicating that the WHO-definitions of obesity based on BMI still do not account for the excess risk of Asians [25].

Present SEP (income-generating work) was significantly inversely associated with diabetes in the multivariate models for men, even after adjustment for SEP from earlier life (body height and education) as confounders, but all the SEP factors contributed to reduce the OR for diabetes for both genders. Migration per ce induces stress and may lead to a rapid "westernization" of lifestyles, and both may increase the risk of disease [45]. This implies that societal factors acting through the life course also should be addressed when studying diabetes causation and ethnic differences in its prevalence [4].

### Strengths and limitations

This study sheds light on the increased susceptibility for diabetes in the first generation of four large ethnic minority groups from Asia living in Europe. The unique opportunity in Scandinavian countries of sampling from population registries with a defined population base and information about demographics of residents has advantages over other sampling techniques [46]. This allowed us to sample well defined ethnic groups and avoid using broad, heterogeneous categorizations of ethnicity [1]. The data collection was performed by the Norwegian Institute of Public Health according to established standards. A wide range of risk factors for diabetes were available, including BMI and three simple and clinically relevant measures of central adiposity, information about physical activity (two measures) and several other potential confounders, not least SEP. In contrast to most studies addressing the differential impact of adiposity on diabetes susceptibility, we were able to adjust for SEP factors across the life course. The inclusion of income-generating work, in addition to education, is not least relevant for immigrants, as education from their country of origin may not be fully recognized in the labour market. For most adults in working age, being without income-generating work, despite the variable's limitations, indicates lower income than if being part of the work force, except for women with husbands with high income.

We are not aware of previous studies that have explored the risk of diabetes related to SEP and adiposity for subjects from Sri Lanka or Vietnam living in Europe. The high diabetes risk among the Vietnamese may easily be underestimated as they are less obese than the other ethnic groups. The sample sizes and the number with diabetes are larger than in many other studies from Europe covering ethnic minority groups, with nearly 900 subjects from Sri Lanka and 1900 Norwegians as the reference population.

The study nevertheless has several limitations, not least due to the cross-sectional design and possible selection biases. Attendance rates in surveys in Norway have fallen markedly during the last few decades [33]. It is difficult to reach first generation immigrants by invitations to studies by mail [1]. However, detailed analyses of the non-attendees have been performed for both studies, and even the prevalence estimates were found to be relatively robust [33]. Furthermore, people attending after one or two reminders reported similar health status and smoking habits as people attending after the first letter of invitation, indicating little selection bias. Although selection biases may be operating, it is unlikely that they could explain the large ethnic differences in diabetes and its risk factors. When studying the associations between disease and risk factors as in this study, the effect of selection bias will be less than when assessing prevalence estimates or population means of risk factors. Furthermore, adjusting for SEP may not be equally valid across different ethnic groups [45]. The impact of these factors within and between ethnic groups on obesity and the risk of type 2 diabetes should be recognised both as structural causes and potential confounders, but are not properly addressed in many studies. Although the effect was in the expected direction, residual confounding may still be operating. The ethnic differences for women, however, were robust, even after adjusting for three SEP factors through the life course (body height, education and income-generating work). Even though validity problems assessing physical activity exist, the dominant finding is the low level of physical activity, especially in the ethnic minority groups. When the majority is sedentary, the association with diabetes will most likely be underestimated. We excluded the Iranians in the analyses of associations due to few cases of diabetes. However, their age-standardized prevalences of know diabetes (women, 4.0% (1.1-6.9), men: 3.3% (1.2-5.4)) were comparable to findings in their country of origin [47], but we found no screening-detected cases in Iranian women. As OGTT was not performed, we have probably underestimated the total diabetes prevalence, and the ORs may be biased towards neutrality.

### Implications

Increased susceptibility for diabetes extends to a wide variety of ethnic groups other than those first described such as American Indians, Aboriginals, South Asians, African descendants or Hispanics, as they meet the obesity epidemic, leaving Europeans and their descendants as the only population group relatively "resistant" to the consequences of obesity [48]. More research is needed to identify and validate the cut-off values of the most clinically relevant adiposity measures to predict future CVD and type 2 diabetes in other ethnic groups than those with European origin [23,25,44]. Adult chronic diseases may be the result of the complex interplay of critical periods, tracking of risk factors through childhood and adulthood and accumulation processes, even acting over generations [4]. Our finding that the increased susceptibility for diabetes for men from Turkey and Vietnam compared with Norwegians disappeared after adjustment for one or more SEP factors (body height, education and income-generating work), indicates that the lower SEP in these ethnic groups partly explains their excess diabetes prevalence, in line with what is found in other studies from Europe [3]. This points to the potential for prevention of diabetes, including broad national policy strategies to reduce social and ethnic inequalities in health [1,49]. The different impact of ethnicity for men and women, indicate that cultural norms and gender roles may also be operating. Nevertheless, the high risk of diabetes by even mild adiposity and the relatively high prevalence of obesity in first generation women from Sri Lanka, Pakistan, Vietnam and Turkey in Norway are worrisome and should alert public health authorities to strengthened actions to prevent obesity in young women due to its potential intergenerational effect. Identification and treatment of gestational diabetes and lifestyle intervention in women with previous gestational diabetes seem to be one rational strategy [50].

## Conclusions

An alarmingly high prevalence of diabetes was found in ethnic minority groups in Oslo born in Sri Lanka and Pakistan, and higher rates compared with Norwegian counterparts were also observed in women from Turkey and Vietnam. In all ethnic minority groups an increased susceptibility for diabetes was observed compared with Norwegians within the same categories of BMI, WC and WHR, highest for Sri Lankans, Pakistanis and Vietnamese, and more for women than men, supporting the need for ethnicity-specific measures of obesity. Ethnic differences in the OR for diabetes were highly significant for all ethnic minority groups compared to Norwegians after adjustment for age and WHR, and persisted for all ethnic minority women and for men from Sri Lanka and Pakistan after further adjustment for SEP from early and later life.

## Abbreviations

UK: United Kingdom; SEP: Socio-economic position; FSG: Fasting serum glucose; NFSG: Non-fasting serum glucose; WC: Waist circumference; WHR: Waist-hip ratio; WSR: Waist-to-stature (body height)-ratio.

## Competing interests

The authors declare that they have no competing interests.

## Authors' contributions

AKJ initiated and was the leader of The Romsås in Motion Study and contributed to the overall leadership and guidance on the development of the paper, in close collaboration with LMD and KIB. GHO and BNK contributed with data from The Immigrant Health Study. LMD did all the analyses, supported by discussions about statistical methods with IMH. LMD prepared the figures. AKJ prepared the first and the revised drafts of the paper. All authors contributed to the discussion of results and to the revisions of the paper. All authors have read and approved the final version.

## Pre-publication history

The pre-publication history for this paper can be accessed here:

http://www.biomedcentral.com/1471-2458/12/150/prepub
